# Radiation-entropy generation by leaf and negentropy build-up of plant as dissipative structure

**DOI:** 10.1140/epje/s10189-026-00555-1

**Published:** 2026-03-11

**Authors:** Shripad P. Mahulikar, Pallavi Rastogi, Aitor Erkoreka

**Affiliations:** 1 Departamento de Ingeniería Energética, Escuela de Ingeniería de Bilbao, University of the Basque Country (EHU), Plaza Torres Quevedo 1, San Mamés, 48013 Bilbao, Pais Vasco Spain; 2 Basque Foundation for Science, IKERBASQUE, Plaza Euskadi 5, 48009 Bilbao, Pais Vasco Spain; 3 Department of Aerospace Engineering, Indian Institute of Technology Bombay, Mumbai, India

## Abstract

Photosynthesis is a simple reaction for any plant, but it has not been achieved artificially due to the difficulty in radiation entropy production by processing PAR (photosynthetically active radiation). In this study, a plant is shown to be a self-organising dissipative structure; since, it’s leaf generates radiation entropy for the existence of plant with possible growth. Entropy-energy ratio (*s*_E_) of PAR released after processing (*s*_E,rel_) much exceeds *s*_E_ of light absorbed, *s*_E,in_ (< < *s*_E,rel_). Plant builds its negentropy $\left| {s_{{{\mathrm{neg}},{\mathrm{rad}}}} } \right|$ by the difference, $s_{{\mathrm{E,rel}}} - s_{{\mathrm{E,in}}}$ , but only a part of this difference can be the source of free energy as plant-growth. The remaining part of $\left| {s_{{{\mathrm{neg}},{\mathrm{rad}}}} } \right|$ is dissipated as the excess payment of negentropy debt, towards mandatory 2nd Law compliance. Change from low *s*_E,in_ to high *s*_E,rel_ by the plant-leaf is amplified by a huge factor, *c*^2^, while determining $\left| {s_{{{\mathrm{neg}},{\mathrm{rad}}}} } \right|$; where, *c* is the speed of light. Therefore, even a small increase in *s*_E,rel_ and small reduction in *s*_E,in_ can significantly increase $\left| {s_{{{\mathrm{neg}},{\mathrm{rad}}}} } \right|$. Thermodynamic performance of photosynthesis depends on the processing level of PAR, $\Pi_{{{\mathrm{E}},{\mathrm{leaf}}}} = \left( {{{s_{{{\mathrm{E}},{\mathrm{rel}}}} } \mathord{\left/ {\vphantom {{s_{{{\mathrm{E}},{\mathrm{rel}}}} } {s_{{{\mathrm{E}},{\mathrm{in}}}} }}} \right. \kern-0pt} {s_{{{\mathrm{E}},{\mathrm{in}}}} }}} \right)$, $\left| {s_{{{\mathrm{neg}},{\mathrm{rad}}}} } \right|$, and is limited by the maximum photosynthetic efficiency in PAR, ${\upeta }_{{{\mathrm{ph}},{\mathrm{PAR}}\_{\mathrm{max}}}}$ (it increases with $\Pi_{{{\mathrm{E}},{\mathrm{leaf}}}}$).

**Graphical Abstract:**

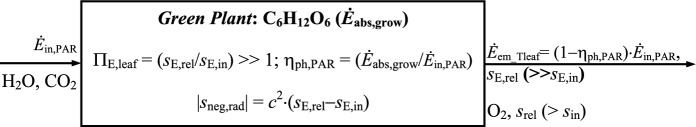

## Introduction to thermodynamics of photosynthesis

Life forms, including plants are systems that are formed and exist far from global thermodynamic equilibrium. In this regime, entropy production is governed by the *Law of Maximum Entropy Production* (LMEP) [1], also known as the Maximum Entropy Production Principle (MEPP) [2]. The *Least Action Principle* (selection of available net path of least resistance to entropy production) when applied to dissipative systems reduces to LMEP/MEPP [3].

### Background on self-organizing dissipative structure (DS) from 1st law and 2nd law

**Fig. 1:**
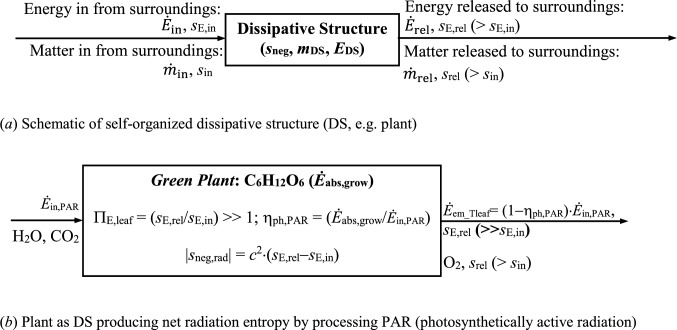
Schematic of net entropy production in the surroundings by self-organized plant as DS. **a** Schematic of self-organized dissipative structure (DS, e.g. plant). **b** Plant as DS producing net radiation entropy by processing PAR (photosynthetically active radiation)

Irreversible processing of mass-energy in-flows taken from the surroundings by a *Dissipative Structure* (*DS*) [4] generates net entropy at rate ($\Delta \dot{S}_{{{\mathrm{gen}},{\mathrm{sur}}}}$) in the surroundings (ref. Table 1 for definitions of scientific terms in this study). These mass-energy interactions maximize the entropy production globally, considering DS and its surroundings together. Self-organizing DS, as schematically illustrated in Fig. 1a, has the following thermodynamic features:

(i) Mass content m_DS_ and energy content E_DS_. Entropies of mass and energy content of DS are, ${\mathrm{S}}_{{{\mathrm{DS}}}} \left( { = {\mathrm{m}}_{{{\mathrm{DS}}}} \cdot {\mathrm{s}}_{{{\mathrm{DS}}}} } \right)$ and ${\mathrm{S}}_{{{\mathrm{E}},{\mathrm{DS}}}} \left( { = {\mathrm{E}}_{{{\mathrm{DS}}}} \cdot {\mathrm{s}}_{{{\mathrm{E}},{\mathrm{DS}}}} } \right)$ ; where, s_DS_ (entropy per unit mass) is entropy density of m_DS_ and s_E,DS_ (entropy per unit energy) is entropy density of E_DS_.
(ii) Mass and energy flow rates taken in by DS from its surroundings are, ${\dot{m}} _{{{\mathrm{in}}}}$ and $\dot{E}_{{{\mathrm{in}}}}$. Entropy in-flow rates associated with ${\dot{m}} _{{{\mathrm{in}}}}$ and $\dot{E}_{{{\mathrm{in}}}}$ are, ${\dot{\mathrm{S}}}_{{{\mathrm{in}}}} \left( { =  {\dot{m}} _{{{\mathrm{in}}}} \cdot {\mathrm{s}}_{{{\mathrm{in}}}} } \right)$ and ${\dot{\mathrm{S}}}_{{{\mathrm{E}},{\mathrm{in}}}} \left( { = \dot{E}_{{{\mathrm{in}}}} \cdot {\mathrm{s}}_{{{\mathrm{E}},{\mathrm{in}}}} } \right)$.
(iii) Mass and energy flow rates released to the surroundings after processing by DS are ${\dot{m}} _{{{\mathrm{rel}}}}$ and $\dot{E}_{{{\mathrm{rel}}}}$. Entropy release rates associated with ${\dot{m}} _{{{\mathrm{rel}}}}$ and $\dot{E}_{{{\mathrm{rel}}}}$ are, ${\dot{\mathrm{S}}}_{{{\mathrm{rel}}}} \left( { =  {\dot{m}} _{{{\mathrm{rel}}}} \cdot {\mathrm{s}}_{{{\mathrm{rel}}}} } \right)$ and ${\dot{\mathrm{S}}}_{{{\mathrm{E}},{\mathrm{rel}}}} \left( { = \dot{E}_{{{\mathrm{rel}}}} \cdot {\mathrm{s}}_{{{\mathrm{E}},{\mathrm{rel}}}} } \right)$.

**Table 1:** Glossary of important thermodynamic terms used in this study

Term	Description
Dissipative structure (DS)	Phrase coined by I. Prigogine [4]: Refers to localised ordering that can exist far from global thermodynamic equilibrium, to increase the global *free energy dissipation* rate
Entropy	Measure of disorder or randomness
Global equilibrium	Zero unbalanced potentials (or driving forces) within an isolated system
Free energy	Energy in a physical system, which can be harnessed for useful mechanical work
Photosynthetically active radiation (PAR)	Range of solar radiation (0.4 to 0.7 μm) that photosynthetic organisms can use for photosynthesis. Energy of photons at shorter wavelengths (< 0.4 μm) can be damaging to the cells and tissues, leading to injury of the leaf. Photons at longer wavelengths (> 0.7 μm) carry insufficient energy for photosynthesis to occur
Law of maximum entropy production	*Isolated System* away from equilibrium will select least resistance path or assemblage of paths out of available paths that minimizes the potential or maximizes the entropy at the fastest rate for given constraints [1]

In-flow rates in DS (e.g. plant), $\dot{E}_{{{\mathrm{in}}}}$ and ${\dot{m}} _{{{\mathrm{in}}}}$, are split in two separate parts, for processing of radiation energy (as in photosynthesis) and matter. By the conservation of mass and energy, DS growth rates are given as;

1 $$  \dot{E}_{{{\mathrm{DS}}}}  = \dot{E}_{{{\mathrm{in}}}}  - \dot{E}_{{{\mathrm{rel}}}} ,\quad{\mathrm{and}}\quad \dot{m}_{{{\mathrm{DS}}}}  = \dot{m}_{{{\mathrm{in}}}}  - \dot{m}_{{{\mathrm{rel}}}}   $$

Absorbed matter/radiation energy, ${\dot{m}} _{{{\mathrm{DS}}}}$ > 0 and $\dot{E}_{{{\mathrm{DS}}}}$  > 0, indicate growth of DS (${\dot{m}} _{{{\mathrm{DS}}}}$ < 0, $\dot{E}_{{{\mathrm{DS}}}}$ < 0, indicate DS-decay). From Eq. (1), the efficiency of using in-flows for DS-growth is given as;

1.1 $$ \begin{aligned}&\eta _{{{\mathrm{grow}},{\mathrm{E}}}}  = {\text{ }}1 - \left( {{{\dot{E}_{{{\mathrm{rel}}}} } \mathord{\left/ {\vphantom {{\dot{E}_{{{\mathrm{rel}}}} } {\dot{E}_{{{\mathrm{in}}}} }}} \right. \kern-\nulldelimiterspace} {\dot{E}_{{{\mathrm{in}}}} }}} \right) = \left( {{{\dot{E}_{{{\mathrm{DS}}}} } \mathord{\left/ {\vphantom {{\dot{E}_{{{\mathrm{DS}}}} } {\dot{E}_{{{\mathrm{in}}}} }}} \right. \kern-\nulldelimiterspace} {\dot{E}_{{{\mathrm{in}}}} }}} \right),{\text{ and }}\\ &\eta _{{{\mathrm{grow}},{\mathrm{m}}}}  = {\text{ }}1 - \left( {{{\dot{m}_{{{\mathrm{rel}}}} } \mathord{\left/ {\vphantom {{\dot{m}_{{{\mathrm{rel}}}} } {\dot{m}_{{{\mathrm{in}}}} }}} \right. \kern-\nulldelimiterspace} {\dot{m}_{{{\mathrm{in}}}} }}} \right) = \left( {{{\dot{m}_{{{\mathrm{DS}}}} } \mathord{\left/ {\vphantom {{\dot{m}_{{{\mathrm{DS}}}} } {\dot{m}_{{{\mathrm{in}}}} }}} \right. \kern-\nulldelimiterspace} {\dot{m}_{{{\mathrm{in}}}} }}} \right)\end{aligned} $$

Thus, ${\upeta }_{{{\mathrm{grow}},{\mathrm{E}}}}$ (= ${\upeta }_{{{\mathrm{ph}},{\mathrm{PAR}}}}$ , for plant-leaf) is the fraction of radiation energy input that is used by DS for its growth; where,

1.1.1 $$\begin{aligned} &0 \, < {\upeta }_{{{\mathrm{grow}},{\mathrm{E}}}} < \, \left( {{\upeta }_{{{\mathrm{grow}},{\mathrm{E}}}} } \right)_{{{\mathrm{max}}}} < { 1},{\text{ and }}\\ &0 < {\upeta }_{{{\mathrm{grow}},{\mathrm{m}}}} < \, \left( {{\upeta }_{{{\mathrm{grow}},{\mathrm{m}}}} } \right)_{{{\mathrm{max}}}} < { 1}, \end{aligned}$$

and for decay: ${\upeta }_{{{\mathrm{grow}},{\mathrm{E}}}} < \, 0$ and ${\upeta }_{{{\mathrm{grow}},{\mathrm{m}}}} < \, 0$.

For 2nd Law compliance in the surroundings, the release flows, $\dot{E}_{{{\mathrm{rel}}}}$ and ${\dot{m}} _{{{\mathrm{rel}}}}$, pay the excess negentropy debt for DS existence, by generating net entropy [5]:

2 $$ \begin{aligned}   \Delta \dot{S}_{{{\mathrm{gen,sur}}}}  =  & \left( {\dot{S}_{{{\mathrm{E,rel}}}}  - \dot{S}_{{{\mathrm{E,in}}}} } \right) + \left( {\dot{S}_{{{\mathrm{rel}}}}  - \dot{S}_{{{\mathrm{in}}}} } \right) \\     =  & {\text{ }}\left( {\dot{E}_{{{\mathrm{rel}}}}  \cdot s_{{{\mathrm{E,rel}}}}  - \dot{E}_{{{\mathrm{in}}}}  \cdot s_{{{\mathrm{E,in}}}} } \right) \\     &  + \left( {\dot{m}_{{{\mathrm{rel}}}}  \cdot s_{{{\mathrm{rel}}}}  - \dot{m}_{{{\mathrm{in}}}}  \cdot s_{{{\mathrm{in}}}} } \right) \ge {\text{ }}0. \\  \end{aligned}   $$

If $\Delta \dot{S}_{{{\mathrm{gen}},{\mathrm{sur}}}} = 0$, then no excess negentropy debt is paid by DS to its surroundings; in this case, $\dot{S}_{{{\mathrm{E}},{\mathrm{rel}}}} = \dot{S}_{{{\mathrm{E}},{\mathrm{in}}}}$, and $\dot{S}_{{{\mathrm{rel}}}} = \dot{S}_{{{\mathrm{in}}}}$. In Eq. (2), the entropies, *S*_E_, *S*, are extensive properties, which are written in terms of the respective intensive properties: *s*_E_ (= entropy-energy ratio, entropy density of energy), *s* (= specific entropy, entropy density of mass). For net radiation entropy production in the surroundings by radiation exchange ($\Delta \dot{S}_{{{\mathrm{rad}},{\mathrm{gen}}}}$), the applicable entropy density is *s*_E_ (used in this study). Inequality in Eq. (2) is separated into two terms for radiation and matter exchanged as;

(using Eq. (1)),

$$ \Delta \dot{S}_{{{\mathrm{rad}},{\mathrm{gen}}}} = \dot{E}_{{{\mathrm{rel}}}} \cdot s_{{{\mathrm{E}},{\mathrm{rel}}}} - \dot{E}_{{{\mathrm{in}}}} \cdot s_{{{\mathrm{E}},{\mathrm{in}}}} = \, \left( {s_{{{\mathrm{E}},{\mathrm{rel}}}} - s_{{{\mathrm{E}},{\mathrm{in}}}} } \right) \cdot \dot{E}_{{{\mathrm{rel}}}} - s_{{{\mathrm{E}},{\mathrm{in}}}} \cdot \dot{E}_{{{\mathrm{DS}}}} \ge \, 0 $$

2.1 $$ \Delta \dot{S}_{{{\mathrm{mat}},{\mathrm{gen}}}} =  {\dot{m}} _{{{\mathrm{rel}}}} \cdot s_{{{\mathrm{rel}}}} -  {\dot{m}} _{{{\mathrm{in}}}} \cdot s_{{{\mathrm{in}}}} \ge \, 0. $$

Dimensionless ratios of exit to inlet entropy densities:

2.2 $$\Pi _{{{\mathrm{E}},{\mathrm{DS}}}} = \left( {{{s_{{{\mathrm{E}},{\mathrm{rel}}}} } \mathord{\left/ {\vphantom {{s_{{{\mathrm{E}},{\mathrm{rel}}}} } {s_{{{\mathrm{E}},{\mathrm{in}}}} }}} \right. \kern-0pt} {s_{{{\mathrm{E}},{\mathrm{in}}}} }}} \right) > > {1},\quad\Pi _{{\mathrm{m}}} = \, \left( {{{s_{{{\mathrm{rel}}}} } \mathord{\left/ {\vphantom {{s_{{{\mathrm{rel}}}} } {s_{{{\mathrm{in}}}} }}} \right. \kern-0pt} {s_{{{\mathrm{in}}}} }}} \right) > > {1}; $$

are the measures/indicators of processing level of in-flows by self-organizing DS. From Eq. (2.1), growth rate of self-organizing DS [$\dot{E}_{{{\mathrm{DS}}}} > 0,\quad  {\dot{m}} _{{{\mathrm{DS}}}} > 0$, ref. Equation (1)] is increased by reducing the release flows for given in-flows as;

2.3 $$ \dot{E}_{{{\mathrm{rel}},{\mathrm{min}}}} < \dot{E}_{{{\mathrm{rel}}}} < \dot{E}_{{{\mathrm{in}}}} ,\quad  {\dot{m}} _{{{\mathrm{rel}},{\mathrm{min}}}} <  {\dot{m}} _{{{\mathrm{rel}}}} <  {\dot{m}} _{{{\mathrm{in}}}} $$

Released mass-energy content can be lower than the in-flows due to processing by DS, to enable DS-growth; but the inequalities in Eq. (2.1) must be satisfied regardless of DS-growth. Therefore, release flows must exceed a minimum threshold, $\dot{E}_{{{\mathrm{rel}},{\mathrm{min}}}}$, ${\dot{m}} _{{{\mathrm{rel}},{\mathrm{min}}}}$; as determined by 2nd Law compliance. From Eq. (2.1): for the case, $\Delta \dot{S}_{{{\mathrm{rad}},{\mathrm{gen}}}} = 0$ (no excess payment of negentropy debt), gives $\dot{E}_{{{\mathrm{rel}},{\mathrm{min}}}} = \, \left( {{{\dot{E}_{{{\mathrm{in}}}} } \mathord{\left/ {\vphantom {{\dot{E}_{{{\mathrm{in}}}} } {\Pi _{{{\mathrm{E}},{\mathrm{DS}}}} }}} \right. \kern-0pt} {\Pi _{{{\mathrm{E}},{\mathrm{DS}}}} }}} \right)$; similarly for $\left( {\Delta \dot{S}_{{{\mathrm{gen}},{\mathrm{sur}}}} } \right)_{{\mathrm{m}}} = 0$, gives ${\dot{m}} _{{{\mathrm{rel}},{\mathrm{min}}}} = \left( { {\dot{m}} _{{{\mathrm{in}}}} /\Pi _{{\mathrm{m}}} } \right)$. Therefore, maximum DS-growth margins (for given in-flows) are, $\Delta \dot{E}_{{{\mathrm{grow}},{\mathrm{max}}}} = \dot{E}_{{{\mathrm{in}}}} - \dot{E}_{{{\mathrm{rel}},{\mathrm{min}}}} = \dot{E}_{{{\mathrm{in}}}} \cdot \left[ {1 - \left( {{1 \mathord{\left/ {\vphantom {1 {\Pi _{{{\mathrm{E}},{\mathrm{DS}}}} }}} \right. \kern-0pt} {\Pi _{{{\mathrm{E}},{\mathrm{DS}}}} }}} \right)} \right]$, $\Delta  {\dot{m}} _{{{\mathrm{grow}},{\mathrm{max}}}} =  {\dot{m}} _{{{\mathrm{in}}}} - {\mathrm{m}}_{{{\mathrm{rel}},{\mathrm{min}}}} =  {\dot{m}} _{{{\mathrm{in}}}} \cdot \left[ {1 - \left( {{1 \mathord{\left/ {\vphantom {1 {\Pi _{{\mathrm{m}}} }}} \right. \kern-0pt} {\Pi _{{\mathrm{m}}} }}} \right)} \right]$. In Eq. (1.1.1), corresponding maximum efficiency of using the in-flows for DS-growth are:

2.3.1 $$ \begin{aligned}   \left( {\eta _{{{\mathrm{grow,E}}}} } \right)_{{\max }}  =  & {\text{ }}1 - \left( {1/\Pi _{{{\mathrm{E,DS}}}} } \right){\text{ and }}\left( {\eta _{{{\mathrm{grow,m}}}} } \right)_{{\max }}  \\     =  & {\text{ }}1 - \left( {1/\Pi _{{\mathrm{m}}} } \right). \\  \end{aligned}  $$

These maximum values of efficiencies increase with higher processing of the mass-energy in-flows by DS, i.e. at higher values of $\Pi _{{{\mathrm{E}},{\mathrm{DS}}}} ,\Pi _{{\mathrm{m}}}$.

Equation (2.1) has 2nd Law compliance inequality for the radiation energy exchanged, which is applicable to a plant-leaf. Following relation is obtained by re-writing $\Delta \dot{S}_{{{\mathrm{rad}},{\mathrm{gen}}}}$ using Eq. (1):

2.4 $$ \left( {s_{{{\mathrm{E}},{\mathrm{rel}}}} - s_{{{\mathrm{E}},{\mathrm{in}}}} } \right) \cdot \dot{E}_{{{\mathrm{rel}}}} = \Delta \dot{S}_{{{\mathrm{rad}},{\mathrm{gen}}}} + s_{{{\mathrm{E}},{\mathrm{in}}}} \cdot \dot{E}_{{{\mathrm{DS}}}} > > \, 0. $$

Satisfying the above inequality is needed for compliance with the following inequalities separately:

(i) Net radiation entropy generated in the surroundings, $\Delta \dot{S}_{{{\mathrm{rad}},{\mathrm{gen}}}} \ge \, 0$ [ref. Equation (2.1)], for 2nd Law compliance in the relatively infinite surroundings of DS. It is the excess negentropy debt paid for sustained DS-existence by radiation entropy generation in the surroundings.
(ii) $s_{{{\mathrm{E}},{\mathrm{in}}}} \cdot \dot{E}_{{{\mathrm{DS}}}}$ ≥ 0, for growth of self-organised DS, by integrating low entropy density content.

Using Eq. (1.1), Eq. (2.4) reduces to,

2.4.1 $$ s_{{\mathrm{E,rel}}} - s_{{\mathrm{E,in}}} = \frac{{\Delta \dot{S}_{{\mathrm{rad,gen}}} }}{{\dot{E}_{{{\mathrm{in}}}} \left( {1 - {\upeta }_{{\mathrm{grow,E}}} } \right)}} + \frac{{s_{{\mathrm{E,in}}} \cdot {\upeta }_{{\mathrm{grow,E}}} }}{{1 - {\upeta }_{{\mathrm{grow,E}}} }} > > 0. $$

Processing level of self-organized DS (e.g. plant) due to the difference, $s_{{{\mathrm{E}},{\mathrm{rel}}}} - s_{{{\mathrm{E}},{\mathrm{in}}}}$, is explained as follows:

(i) More growth of self-organized DS is when, ${\upeta }_{{\mathrm{grow,E}}}$ is high, but *s*_E,in_ (integrated with DS) is low. Photosynthetic efficiency of photosynthetically active radiation [= ${\upeta }_{{{\mathrm{ph}},{\mathrm{PAR}}}}$ , shown later in Eq. (3.1)] is ${\upeta }_{{{\mathrm{grow}},{\mathrm{E}}}}$.
(ii) For given processing level [fixed left hand-side in Eq. (2.4.1)], growth of self-organized DS is by reducing $\Delta \dot{S}_{{{\mathrm{rad}},{\mathrm{gen}}}}$ subject to, $\Delta \dot{S}_{{{\mathrm{rad}},{\mathrm{gen}}}} > 0$.

### Motivation, objectives and scope

Plant with growth potential is a self-organized dissipative structure (DS), just as any other life-form. For its existence and growth with mandatory 2nd Law compliance [Eq. (2.1)], its leaves generate radiation entropy in the surroundings. Radiation entropy generation is by using part of the incident energy in Photosynthetically Active Radiation (PAR), which is processed by the plant-leaf but not absorbed for plant-growth. The PAR is from 0.4–0.7 µm [6], which has significant overlap with the visible spectrum, 0.38–0.75 μm. The PAR includes, λ_peak_ = 0.5 μm (wavelength of peak emission from Wien’s Displacement Law, within green colour), corresponding to the Sun’s surface temperature, *T*_sun_ ≈ 5772 K.

This theoretical research thermodynamically studies the photochemical reaction that all plants accomplish naturally with ease, but so far has not been achieved artificially (even at laboratory-scale). Difficulty is the formation of highly ordered C_6_H_12_O_6_ molecules having low entropy density (with several covalent bonds), using energy of sunlight in PAR for radiation entropy generation in the surroundings. Radiation at high entropy density released (*s*_E,rel_) at the plant-leaf’s temperature (to generate entropy in surroundings) relative to the low entropy density of PAR absorbed (*s*_E,in_), is by the processing of PAR. In photosynthesis, energy in PAR is used to break the bonds in CO_2_ and liquid water. Atoms are then recombined into O_2_ and C_6_H_12_O_6_ in which, radiation energy is stored as potential energy in the formation of covalent bonds of glucose. Hence, chemical energy stored in the bonds is a measure of glucose production. This light to chemical energy conversion in photosynthesis annually results in the world-wide storage of ~ 3 × 10^21^ J of energy in glucose, C_6_H_12_O_6_. But world’s net population is increasing and economic development is diminishing the land resources available for agricultural cultivation. Therefore, doubling of agricultural productivity is needed by the end of this century for sustainability [7]. Photosynthetic efficiency [${\upeta }_{{{\mathrm{ph}},{\mathrm{PAR}}}}$ , Eq. (1.1)] is a key parameter determining the thermodynamic performance of a plant-leaf. It is the fraction of radiation energy in PAR intake that is absorbed for plant-growth by producing chemical energy, which is stored in the bonds of glucose [8]. Hence, an insightful study of light-to-chemical energy conversion [9] using the thermodynamic efficiency of photosynthesis ${\upeta }_{{{\mathrm{th}} - {\mathrm{ph}}}}$ [mathematically defined later in Eq. (3.1.4)] and ${\upeta }_{{{\mathrm{ph}},{\mathrm{PAR}}}}$, is needed. Re-visit to the application of 1st and 2nd laws can give better insights into the mechanism of photosynthesis. Energy balance equation (from 1st Law) and entropy balance equation (from 2nd Law) are strongly coupled in photosynthesis, which is modelled by combining them [Eq. (2.4.1)].

Early studies on the thermodynamics of photosynthesis were based on the Carnot cycle model [10], which concluded that ${\upeta }_{{{\mathrm{th}} - {\mathrm{ph}}}}$ is inherently limited. Bolton & Hall [11] theoretically estimated that the maximum thermodynamic efficiency of photosynthesis, ${\upeta }_{{{\mathrm{th}} - {\mathrm{ph}},{\mathrm{max}}}}$  ~ 13%, in green-plants that release O_2_ by inducing the oxidation of water in bright sunlight. Parson [12] argued that the Carnot cycle model does not apply to photo-chemical process, because ${\upeta }_{{{\mathrm{th}} - {\mathrm{ph}}}}$ is determined by the chemical kinetics of species in reaction. Jennings et al. [13] supported Parson [12] on the invalidity of Carnot cycle model to describe ${\upeta }_{{{\mathrm{th}} - {\mathrm{ph}}}}$, but concluded that 2nd Law is violated. This conclusion was soon refuted by Lavergne [14], who discussed a photochemical energy transducer as a model for photosynthesis that must necessarily satisfy 2nd Law. Knox & Parson [15] theoretically proved that 2nd Law is not violated in a photochemical energy conversion, regardless of the value of photosynthetic efficiency, η_ph_. Literature indicates that there is a lack of conceptual clarity on the non-equilibrium thermodynamic basis of η_ph_. It is addressed in this theoretical study by modelling plant as self-organising DS and by using 1st Law (conservation principle) and 2nd Law.

## Plant as self-organizing dissipative structure (DS)

Incident sunlight on a plant-leaf’s surface has two parts, $\dot{E}_{{{\mathrm{inc}}}} = \dot{E}_{{{\mathrm{inc}},{\mathrm{PAR}}}} + \dot{E}_{{{\mathrm{inc}},{\mathrm{PnAR}}}}$; where, $\dot{E}_{{{\mathrm{inc}},{\mathrm{PnAR}}}}$ is Photosynthetically non-Active Radiation (PnAR). Distribution of incident sunlight in PAR on a plant-leaf is as follows [16]: $\dot{E}_{{{\mathrm{inc}},{\mathrm{PAR}}}} = \dot{E}_{{{\mathbf{in}},{\mathbf{PAR}}}} + \dot{E}_{{{\mathrm{refl}},{\mathrm{no}}\_{\mathrm{proc}}}} + \dot{E}_{{{\mathrm{trans}},{\mathrm{no}}\_{\mathrm{proc}}}} = \, \left( {\dot{E}_{{{\mathbf{abs}},{\mathbf{grow}}}} + \dot{E}_{{{\mathbf{em}}\_{\mathbf{Tleaf}}}} } \right) \, + \dot{E}_{{{\mathrm{refl}},{\mathrm{no}}\_{\mathrm{proc}}}} + \dot{E}_{{{\mathrm{trans}},{\mathrm{no}}\_{\mathrm{proc}}}}$. In this equation, $\dot{E}_{{{\mathrm{in}},{\mathrm{PAR}}}}$ is the energy intake by the plant-leaf, part of which is absorbed for plant-growth $\dot{E} {_{{{\mathrm{abs}},{\mathrm{grow}}}} }$ and the remaining part is emitted at the leaf temperature (*T*_leaf_) after processing, $\dot{E}_{{{\mathrm{em}}\_{\mathrm{Tleaf}}}}$. Plant-leaf typically takes in ~ 85% of incident sunlight in PAR, i.e. plant-leaf’s absorptivity, ${\upalpha }_{{{\mathrm{leaf}},{\mathrm{PAR}}}} = \, \left( {{{\dot{E}_{{{\mathrm{in}},{\mathrm{PAR}}}} } \mathord{\left/ {\vphantom {{\dot{E}_{{{\mathrm{in}},{\mathrm{PAR}}}} } {\dot{E}_{{{\mathrm{inc}},{\mathrm{PAR}}}} }}} \right. \kern-0pt} {\dot{E}_{{{\mathrm{inc}},{\mathrm{PAR}}}} }}} \right) \approx 0.{85}$ [17]; which differs from the total absorptivity, ${\upalpha }_{{{\mathrm{leaf}},{\mathrm{tot}}}} = {{\left( {\dot{E}_{{{\mathrm{in}},{\mathrm{PAR}}}} + \dot{E}_{{{\mathrm{abs}},{\mathrm{PnAR}}}} } \right)} \mathord{\left/ {\vphantom {{\left( {\dot{E}_{{{\mathrm{in}},{\mathrm{PAR}}}} + \dot{E}_{{{\mathrm{abs}},{\mathrm{PnAR}}}} } \right)} {\left( {\dot{E}_{{{\mathrm{inc}},{\mathrm{PAR}}}} + \dot{E}_{{{\mathrm{inc}},{\mathrm{PnAR}}}} } \right)}}} \right. \kern-0pt} {\left( {\dot{E}_{{{\mathrm{inc}},{\mathrm{PAR}}}} + \dot{E}_{{{\mathrm{inc}},{\mathrm{PnAR}}}} } \right)}}$. A small percentage of $\dot{E}_{{{\mathrm{inc}},{\mathrm{PAR}}}}$ (~ 10%) is reflected without processing ($\dot{E}_{{{\mathrm{refl}},{\mathrm{no}}\_{\mathrm{proc}}}}$ ) and the remaining 5% is transmitted without processing,$\dot{E}_{{{\mathrm{trans}},{\mathrm{no}}\_{\mathrm{proc}}}}$ . Spectral absorptivity, α_λ_, is the highest for blue and red light ~ 0.80–0.95 and lower for green light ~ 0.5–0.8 [18].

By energy conservation, PAR taken in by the plant-leaf is distributed as follows:

3 $$ \dot{E}_{{{\mathbf{in}},{\mathbf{PAR}}}} = \dot{E}_{{{\mathbf{abs}},{\mathbf{grow}}}} + \dot{E}_{{{\mathbf{em}}\_{\mathbf{Tleaf}}}} ; $$

where, $\dot{E}_{{{\mathrm{in}},{\mathrm{PAR}}}} = \dot{E}_{{{\mathrm{in}}}}$, in Eq. (1.1). Part of $\dot{E}_{{{\mathrm{in}},{\mathrm{PAR}}}}$ (determined by photosynthetic efficiency) is absorbed for plant-growth $\dot{E}{_{{{\mathrm{abs}},{\mathrm{grow}}}} }$ [= *Ė*_DS_, in Eq. (1.1)] = ${\upeta }_{{{\mathrm{ph}},{\mathrm{PAR}}}} \cdot \dot{E}_{{{\mathrm{in}},{\mathrm{PAR}}}}$. The ${\upeta }_{{{\mathrm{ph}},{\mathrm{PAR}}}}$ is the fraction of sunlight in PAR that is taken in by the plant-leaf and absorbed for plant-growth (by storing in covalent bonds of glucose). From Eq. (1.1),

3.1 $$  \begin{aligned}   \eta _{{{\mathrm{grow}},{\mathrm{E}}}}  =  & \eta _{{{\mathrm{ph}},{\mathrm{PAR}}}}  = \left( {\dot{E}_{{{\mathrm{abs}},{\mathrm{grow}}}} /\dot{E}_{{{\mathrm{in}},{\mathrm{PAR}}}} } \right) \\     =  & {\mathrm{1}} - \left( {\dot{E}_{{{\mathrm{em}}\_{\mathrm{Tleaf}}}} /\dot{E}_{{{\mathrm{in}},{\mathrm{PAR}}}} } \right). \\  \end{aligned}  $$

Photosynthetic efficiency based on fraction of $\dot{E}_{{{\mathrm{inc}},{\mathrm{PAR}}}}$ used for plant-growth (differs from ${\upeta }_{{{\mathrm{ph}},{\mathrm{PAR}}}}$) is given as, ${\upeta }_{{{\mathrm{ph}},{\mathrm{inc}}}} = \left( {{{\dot{E}_{{{\mathrm{abs}},{\mathrm{grow}}}} } \mathord{\left/ {\vphantom {{\dot{E}_{{{\mathrm{abs}},{\mathrm{grow}}}} } {\dot{E}_{{{\mathrm{inc}},{\mathrm{PAR}}}} }}} \right. \kern-0pt} {\dot{E}_{{{\mathrm{inc}},{\mathrm{PAR}}}} }}} \right) = \left( {{{\dot{E}_{{{\mathrm{abs}},{\mathrm{grow}}}} } \mathord{\left/ {\vphantom {{\dot{E}_{{{\mathrm{abs}},{\mathrm{grow}}}} } {\dot{E}_{{{\mathrm{in}},{\mathrm{PAR}}}} }}} \right. \kern-0pt} {\dot{E}_{{{\mathrm{in}},{\mathrm{PAR}}}} }}} \right)\left( {{{\dot{E}_{{{\mathrm{in}},{\mathrm{PAR}}}} } \mathord{\left/ {\vphantom {{\dot{E}_{{{\mathrm{in}},{\mathrm{PAR}}}} } {\dot{E}_{{{\mathrm{inc}},{\mathrm{PAR}}}} }}} \right. \kern-0pt} {\dot{E}_{{{\mathrm{inc}},{\mathrm{PAR}}}} }}} \right)$. From Eq. (3.1), ${\upeta }_{{{\mathrm{ph}},{\mathrm{inc}}}} = {\upeta }_{{{\mathrm{ph}},{\mathrm{PAR}}}} \cdot {\upalpha }_{{{\mathrm{leaf}},{\mathrm{PAR}}}} ;{\text{ thus}},\;{\upeta }_{{{\mathrm{ph}},{\mathrm{inc}}}} < {\upeta }_{{{\mathrm{ph}},{\mathrm{PAR}}}}$ (since, α_leaf,PAR_ ~ 0.85).

Plant is an *open thermodynamic system*, which exchanges radiation and matter with its surroundings; ref. Figure 1b. Endothermic reaction in a plant absorbing $\dot{E}_{{{\mathrm{in}},{\mathrm{PAR}}}}$ [in Eq. (3)] for the synthesis of glucose is,

3.1.1 $$ {\mathrm{6CO}}_{{2}} + {\text{ 6H}}_{{2}} {\text{O }} + \dot{E}_{{{\mathrm{in}},{\mathrm{PAR}}}} \to {\mathrm{C}}_{{6}} {\mathrm{H}}_{{{12}}} {\mathrm{O}}_{{6}} + {\text{ 6O}}_{{2}} + \dot{E}_{{{\mathrm{em}}\_{\mathrm{Tleaf}}}} \left( { < \dot{E}_{{{\mathrm{in}},{\mathrm{PAR}}}} } \right). $$

By photosynthesis, $\dot{E}_{{{\mathrm{in}},{\mathrm{PAR}}}}$ is converted into chemical energy (*Ė*_abs,grow_) stored in the bonds of glucose, C_6_H_12_O_6_. Waste matter O_2_ and grey body radiation *Ė*_em_Tleaf_ are released to the surroundings at higher entropy densities, than the intakes. Therefore, production of glucose retained by the plant for its growth correlates with the release of O_2_ (at relatively higher specific entropy, *s*_O2_) [19] and the release of grey body radiation, *Ė*_em_Tleaf_ (at *s*_E,rel_ >  > *s*_E,in_). Glucose has lower specific entropy due to part of absorbed PAR at low *s*_E,in_ stored in its bonds, which is retained by the plant. In Eq. (3.1.1), *Ė*_em_Tleaf_ [= *Ė*_rel_, in Eq. (1.1)], is thermal radiation at *T*_leaf_, i.e. it includes PAR and is mostly in PnAR (mostly at wavelengths longer than PAR). It is distributed as grey-body radiation over the full electromagnetic spectrum (λ = 0 − ∞ μm) as per Planck’s Law:

3.1.2 $$ \dot{E}_{{{\mathrm{em}}\_{\mathrm{Tleaf}}}} = \, \left( {{1} - {\upeta }_{{{\mathrm{ph}},{\mathrm{PAR}}}} } \right) \cdot \dot{E}_{{{\mathrm{in}},{\mathrm{PAR}}}} = A_{{{\mathrm{leaf}}}} \cdot {{\epsilon}}_{{{\mathrm{leaf}}}} \cdot {\upsigma } \cdot T_{{{\mathrm{leaf}}}}^{4} $$

In Eq. (3.1.2):

(i) *Ė*_em_Tleaf_ (< $\dot{E}_{{{\mathrm{in}},{\mathrm{PAR}}}}$) is the re-emission of PAR processed by plant-leaf at *s*_E,rel_ (> > *s*_E,in_);
(ii) *A*_leaf_ is the surface area of plant-leaf;
(iii) ε_leaf_ is the emissivity of plant-leaf’s surface, which is high (> 0.9) for semi-transparent fresh leaves that have water content [16];
(iv) σ (= 5.6704 × 10^−8^ W/m^2^ K^4^) is the Stephan-Boltzmann constant.

### 2nd law compliance in photosynthesis

From Eq. (2.3.1), the upper limit for plant-growth based on processing of PAR and 2nd Law compliance is given by the maximum photosynthetic efficiency,

3.1.3 $$ {\upeta }_{{{\mathrm{ph}},{\mathrm{PAR}} - {\mathrm{max}}}} = \left( {{\upeta }_{{{\mathrm{grow}},{\mathrm{E}}}} } \right)_{{{\mathrm{max}}}} = { 1} - \left( {{1}/\Pi _{{{\mathrm{E}},{\mathrm{leaf}}}} } \right) \, < { 1}. $$

At ${\upeta }_{{{\mathrm{ph}},{\mathrm{PAR}}}} = {\upeta }_{{{\mathrm{ph}},{\mathrm{PAR}} - {\mathrm{max}}}} ,\quad \dot{E}_{{{\mathrm{em}}\_{\mathrm{Tleaf}}}} = \dot{E}_{{{\mathrm{em}}\_{\mathrm{Tleaf}},{\mathrm{min}}}}$, i.e. $\dot{E}_{{{\mathrm{abs}},{\mathrm{grow}}}} = \dot{E}_{{{\mathrm{abs}},{\mathrm{grow}}\_{\mathrm{max}}}}$ for given $\dot{E}_{{{\mathrm{in}},{\mathrm{PAR}}}}$. Processing level of PAR by plant-leaf is, $\Pi _{{{\mathrm{E}},{\mathrm{leaf}}}} = \, \left( {{{s_{{{\mathrm{E}},{\mathrm{rel}}}} } \mathord{\left/ {\vphantom {{s_{{{\mathrm{E}},{\mathrm{in}}}} } {s_{{{\mathrm{E}},{\mathrm{rel}}}} }}} \right. \kern-0pt} {s_{{{\mathrm{E}},{\mathrm{in}}}} }}} \right)$, ref. Eq. (2.2); therefore, for higher η_ph,PAR-max_ (more self-organization with growth), Π_E,leaf_ must be increased. Equation (3.1.3) gives the upper limit for DS-growth, (η_grow,E_)_max_ [Eq. (2.3.1)], based on 2nd Law compliance. Thus, photosynthetic efficiency, ${\upeta }_{{{\mathrm{ph}},{\mathrm{PAR}}}}$, is inherently limited by the plant-leaf’s ability to process PAR for generating radiation entropy in the surroundings.

From 2nd Law analyses of photosynthesis [20], plant physiologists find ways to increase ${\upeta }_{{{\mathrm{ph}},{\mathrm{PAR}}}}$ for given ${\upeta }_{{{\mathrm{th}} - {\mathrm{ph}}}}$ (= thermodynamic efficiency of photosynthesis) [21]. It is defined as, ${\upeta }_{{{\mathrm{th}} - {\mathrm{ph}}}}$ = (chemical energy stored in bonds of C_6_H_12_O_6_)/[energy of PAR intake that is absorbed for growth, $\dot{E}_{{{\mathrm{abs}},{\mathrm{grow}}}} \left( { = {\upeta }_{{{\mathrm{ph}},{\mathrm{PAR}}}} \cdot \dot{E}_{{{\mathrm{in}},{\mathrm{PAR}}}} } \right)$]. Therefore,

3.1.4 $$ \left( {{\text{chemical energy stored in bonds of C}}_{{6}} {\mathrm{H}}_{{{12}}} {\mathrm{O}}_{{6}} } \right) \, = \, ({\upeta }_{{{\mathrm{ph}},{\mathrm{PAR}}}} \cdot \dot{E}_{{{\mathrm{in}},{\mathrm{PAR}}}} ) \cdot {\upeta }_{{{\mathrm{th}} - {\mathrm{ph}}}} . $$

Part of, *Ė*_abs,grow_ (= ${\upeta }_{{{\mathrm{ph}},{\mathrm{PAR}}}} \cdot \dot{E}_{{{\mathrm{in}},{\mathrm{PAR}}}}$) is stored in the bonds of C_6_H_12_O_6_ [22], which when broken releases free energy (used by plant for its growth, to do work e.g. against Earth’s gravity). From Eq. (3.1.4), increase in chemical energy stored in the bonds of C_6_H_12_O_6_, i.e. more sugar production is achieved by increasing ${\upeta }_{{{\mathrm{ph}},{\mathrm{PAR}}}}$ (for given ${\upeta }_{{{\mathrm{th}} - {\mathrm{ph}}}}$). Using Eq. (2.4) for the photochemical reaction given by Eq. (3.1.1),

3.2 $$\begin{aligned} &\left( {s_{{{\mathrm{E}},{\mathrm{rel}}}} - s_{{{\mathrm{E}},{\mathrm{in}}}} } \right) \cdot \dot{E}_{{{\mathrm{em}}\_{\mathrm{Tleaf}}}} \\ &\quad = \Delta \dot{S}_{{{\mathrm{rad}},{\mathrm{gen}}}} + s_{{{\mathrm{E}},{\mathrm{in}}}} \cdot \dot{E}_{{{\mathrm{abs}},{\mathrm{grow}}}} > > \, 0. \end{aligned}$$

Following from above are the two additional inequalities: (i) for self-organized plant to grow, *s*_E,in_⋅*Ė*_abs,grow_ > 0; (ii) for mandatory 2nd Law compliance, net radiation entropy generated by the plant-leaf in its surroundings, $\Delta \dot{S}_{{{\mathrm{rad}},{\mathrm{gen}}}}$ > 0. Processing of PAR by the plant-leaf (*s*_E,rel_ >  > *s*_E,in_) generates radiation entropy in the surroundings, $\Delta \dot{S}_{{{\mathrm{rad}},{\mathrm{gen}}}}$, for the mandatory 2nd Law compliance. It is the available path of least resistance in LMEP [1] (least action path in dissipative system [3]), to maximise entropy production in the surroundings.

Equation (3.2) is re-written as,

3.2.1 $$ s_{{{\mathrm{E}},{\mathrm{rel}}}} - s_{{{\mathrm{E}},{\mathrm{in}}}} = \frac{{\Delta \dot{S}_{{{\mathrm{rad}},{\mathrm{gen}}}} }}{{\dot{E}_{{{\mathrm{em}}\_{\mathrm{Tleaf}}}} }} + s_{{{\mathrm{E}},{\mathrm{in}}}} \cdot \frac{{{\upeta }_{{{\mathrm{ph}},{\mathrm{PAR}}}} }}{{1 - {\upeta }_{{{\mathrm{ph}},{\mathrm{PAR}}}} }} > > \, 0. $$

Intensity of sunlight in PAR reduces enroute from the Sun’s surface to Earth’s surface (*Ė*_in,PAR_), but its high quality as measured by its low entropy density (low *s*_E,in_) remains unchanged. High entropy density difference on the left-hand side is negentropy build-up of plant [shown later in Eq. (4.2.1)], due to processing of PAR. From Eq. (3.2), dimensionless net radiation entropy generated by the plant-leaf in its surroundings is given as,

3.2.2 $$\begin{aligned}\Pi _{{{\mathrm{Srad}},{\mathrm{gen}}}}& = \frac{{\Delta \dot{S}_{{{\mathrm{rad}},{\mathrm{gen}}}} }}{{s_{{{\mathrm{E}},{\mathrm{in}}}} \cdot \dot{E}_{{{\mathrm{abs}},{\mathrm{grow}}}} }} \\ &=({\Pi _{{ {{\mathrm{E}},{\mathrm{leaf}}}}}}-1) \cdot [( {{1}/{\upeta }_{{{\mathrm{ph}},{\mathrm{PAR}}}})-1 } ] - {1 } \ge \, 0.\end{aligned} $$

**Fig. 2:**
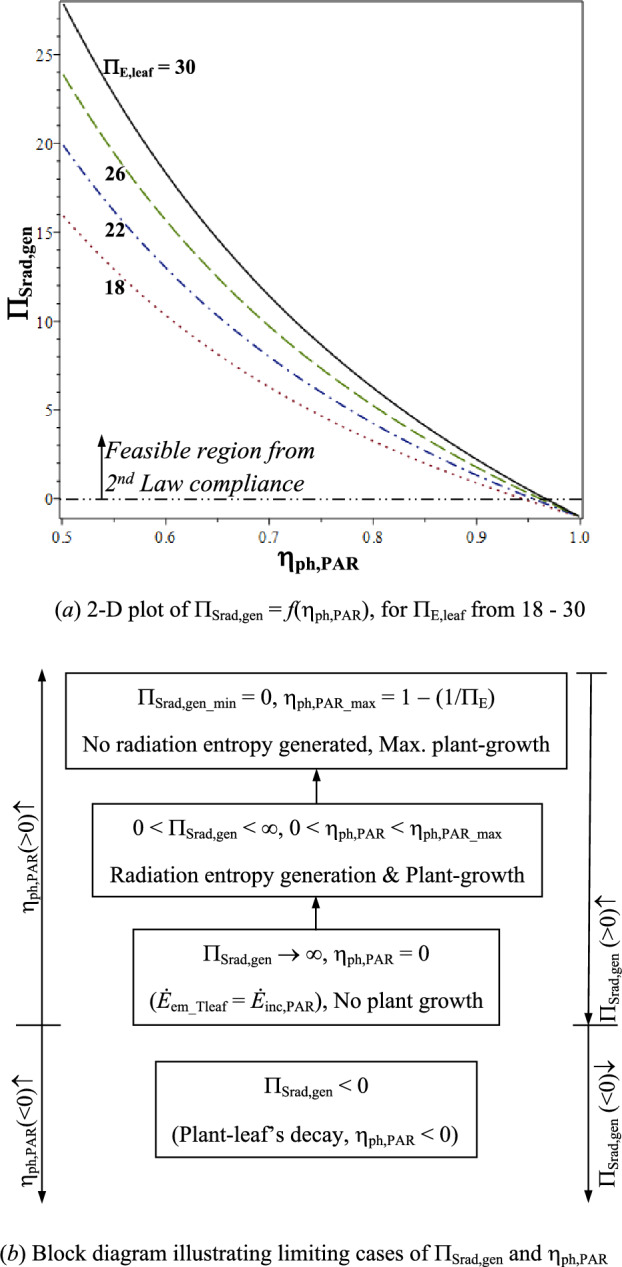
Dimensionless radiation entropy generated by the plant-leaf, $\Pi _{{{\mathrm{Srad}},{\mathrm{gen}}}} = f({\upeta }_{{{\mathrm{ph}},{\mathrm{PAR}}}} ,\Pi _{{{\mathrm{E}},{\mathrm{leaf}}}} )$. **a** 2-D plot of $\Pi _{{{\mathrm{Srad}},{\mathrm{gen}}}} = f({\upeta }_{{{\mathrm{ph}},{\mathrm{PAR}}}} )$, for $\Pi _{{{\mathrm{E}},{\mathrm{leaf}}}}$ from 18 to 30. **b** Block diagram illustrating limiting cases of $\Pi _{{{\mathrm{Srad}},{\mathrm{gen}}}}$ and ${\upeta }_{{{\mathrm{ph}},{\mathrm{PAR}}}}$

Net radiation entropy generated in the surroundings per unit radiation entropy absorbed for plant-growth is, $\Pi_{{{\mathrm{Srad}},{\mathrm{gen}}}}$  ≥ 0, for mandatory 2nd Law compliance regardless of the value of ${\upeta }_{{{\mathrm{ph}},{\mathrm{PAR}}}}$. From Eq. (3.2.2), with increase in $\Pi_{{{\mathrm{E}},{\mathrm{leaf}}}}$ (processing level), $\Pi_{{{\mathrm{Srad}},{\mathrm{gen}}}}$ increases for given ${\upeta }_{{{\mathrm{ph}},{\mathrm{PAR}}}}$. Figure 2a shows, $\Pi_{{{\mathrm{Srad}},{\mathrm{gen}}}} = f\left( {\Pi_{{{\mathrm{E}},{\mathrm{leaf}}}} ,{\upeta }_{{{\mathrm{ph}},{\mathrm{PAR}}}} } \right)$, which decreases with increasing ${\upeta }_{{{\mathrm{ph}},{\mathrm{PAR}}}}$, for given Π_E,leaf_ (from 18 to 30). Therefore, plant-growth is by reducing net radiation entropy generated [ref. Equation (2.4.1)] subject to 2nd Law compliance, i.e. region above, $\Pi_{{{\mathrm{Srad}},{\mathrm{gen}}}}$  ≥ 0 in Fig. 2a, is feasible. Rearranging Eq. (3.2.2),

3.2.3 $$\begin{aligned} {\upeta }_{{{\mathrm{ph}},{\mathrm{PAR}}}} {=}& [{ 1} {-} (1/{\Pi_{\mathrm{E,leaf}}})]/[1{+}(\Pi_{Srad, gen}/{\Pi_{E, leaf}})]\\ & < {\upeta }_{{{\mathrm{ph}},{\mathrm{PAR}} {-} {\mathrm{max}}}} , \end{aligned} $$

thus, ${\upeta }_{{{\mathrm{ph}},{\mathrm{PAR}}}}$ increases with increasing Π_E,leaf_, for given Π_Srad,gen_. Relation between Π_Srad,gen_ and ${\upeta }_{{{\mathrm{ph}},{\mathrm{PAR}}}}$, has the following two limiting cases [ref. block diagram in Fig. 2b]:

(i) Upper limit to plant-growth (zero net radiation entropy generation in surroundings) is the intercept on ${\upeta }_{{{\mathrm{ph}},{\mathrm{PAR}}}}$ (*X*) axis, ${\upeta }_{{{\mathrm{ph}},{\mathrm{PAR}}}} \left( {\Pi _{{{\mathrm{Srad}},{\mathrm{gen}}\_{\mathrm{min}}}} = 0} \right) = {\upeta }_{{{\mathrm{ph}},{\mathrm{PAR}}\_{\mathrm{max}}}}$. It is given by Eq. (3.1.3), which is a special case of Eq. (3.2.3).
(ii) Maximum net radiation entropy generation in the surroundings (no plant-growth) is for, $\Pi _{{{\mathrm{Srad}},{\mathrm{gen}}\_{\mathrm{max}}}} \to \infty$.

Leaf contributes to plant-growth (${\upeta }_{{{\mathrm{ph}},{\mathrm{PAR}}}}$ > 0) when, $\Pi _{{{\mathrm{Srad}},{\mathrm{gen}}}}$ is finite; which is characterised by high processing level of PAR (high Π_E,leaf_). Plant-leaf decays (${\upeta }_{{{\mathrm{ph}},{\mathrm{PAR}}}}$ < 0) when, $\Pi _{{{\mathrm{Srad}},{\mathrm{gen}}}}\,<\,0$, after losing its ability to process PAR (low Π_E,leaf_).

Heat is generated in the plant-leaf by −

(i) processing of *Ė*_in,PAR_ in the plant-leaf due to metabolism, which coverts it in to chemical energy stored in the bonds of C_6_H_12_O_6_;
(ii) absorption of thermal radiation (primarily in infrared spectrum) at wavelengths longer than PAR [23].

For non-transpirating plant-leaf [24], *T*_leaf_ is a bit higher than the local ambient temperature (*T*_a_) and (*T*_leaf_ − *T*_a_) ≠ *f*(*T*_a_), i.e. *T*_leaf_ increases linearly with *T*_a_ [25]. Heat loss from plant-leaf to its surroundings by convection and radiation is at rate (which lowers *T*_leaf_ & reduces disorder content in leaf): $q_{{{\mathrm{HT}}}} = A_{{{\mathrm{leaf}}}} \cdot [h \cdot (T_{{{\mathrm{leaf}}}} - T_{{\mathrm{a}}} ) \, + {{\epsilon}}_{{{\mathrm{leaf}}}} \cdot {\upsigma }\left( {T_{{{\mathrm{leaf}}}}^{4} - T_{{\mathrm{a}}}^{4} } \right)$]. Total irreversible loss in processing incident light in PAR can be as high as 97% [26] and it increases with *T*_leaf_ (due to reduction in processing ability of plant-leaf).

### *Negentropy of plant as dissipative structure* (*DS*) *generating radiation entropy*

Link between information collected by a system from its surroundings for sustained existence was defined as the negative of its entropy reduction by Shannon [27]. Energy alone cannot sustain dissipative structure (DS), ref. Schroedinger [28] (1945), who noted that life maintains its ordered state by “feeding” on negative entropy (negentropy), by taking order from its environment and dissipating disorder. Elitzur [29] noted that an organism (e.g. plant) must have order relative to its environment, by processing information gained from its surroundings.

Self-organization (as measured by negentropy) in plant as DS is by the continuous net in-flux of negative radiation entropy density. Processing of energy and/or mass for the release to surroundings at much higher entropy density than the in-flows, Π_E,DS_ >> 1 [Eq. (2.2)], Π_E,leaf_ >> 1 [Eq. (3.1.3)], is the net in-flux of negative entropy density in a plant as a self-organizing DS. Net in-flux of negative entropy density is needed to at least balance the rise in entropy that inevitably accompanies the irreversible processing inside the plant and also for increasing its self-organization. Mahulikar & Herwig [30] re-defined Schroedinger’s [28] notion of negentropy (*s*_neg_) as the specific entropy contrast of DS relative to its release (immediate surroundings of DS):

4 $$ s_{{{\mathrm{neg}}}} = s_{{{\mathrm{DS}}}} - s_{{{\mathrm{rel}}}} ;{\text{ where}},s_{{{\mathrm{DS}}}} < < s_{{{\mathrm{rel}}}} \Rightarrow s_{{{\mathrm{neg}}}} < < \, 0. $$

From Eq. (2.4.1), mass-energy content of a self-organized DS (*s*_DS_) is made up of processed in-flows with low entropy density (*s*_E,in_, *s*_in_), because higher entropy density content (*s*_rel_) is released to the surroundings. From Eqs. (3.1.1) and (3.2.2), higher entropy density mass-energy content (O_2_, *E*_em_Tleaf_) is released; thus,

4.1 $$ s_{{{\mathrm{in}}}} \to s_{{{\mathrm{DS}}}} < < s_{{{\mathrm{rel}}}} ,{\text{ and }}s_{{{\mathrm{E}},{\mathrm{in}}}} \to s_{{{\mathrm{E}},{\mathrm{DS}}}} < < s_{{{\mathrm{E}},{\mathrm{rel}}}} . $$

Negentropy production (for self-organization) and its link to possible growth of DS (e.g. plant) is a special case of LMEP [1], applicable to DS existence. Formation of complex glucose molecules with large amount of information (large number of bonds) contributes to the negentropy build-up of plant, referred as negative entropy production [13]. Negentropy build-up by the decrease in entropy in building C_6_H_12_O_6_ molecules [31] in the plant is made possible by the simultaneous release of *Ė*_em_Tleaf_ [Eq. (3.1.2)] at high *s*_E,rel_ to surroundings (compensation for mandatory 2nd Law compliance).

Specific entropy per unit mass, *s*_DS_ (entropy-density of mass, SI units [J/kg-K]), relates with entropy-by-energy ratio, *s*_E,DS_ (entropy-density of energy, SI units [K^−1^]) as; *s*_E,DS_ = (*s*_DS_/*c*^2^), where, *c* (= 2.998 × 10^8^ m/s) is the speed of light. From Eq. (4), negentropy of plant as DS, which exists as matter by the exchange of radiation energy and matter with surroundings is,

4.2 $$ \begin{aligned} s_{{{\mathrm{neg}}}} &= s_{{{\mathrm{neg}},{\mathrm{rad}}}} + s_{{{\mathrm{neg}},{\mathrm{mat}}}} =  c^{{2}} \cdot \left( {s_{{{\mathrm{E}},{\mathrm{DS}}}} - s_{{{\mathrm{E}},{\mathrm{rel}}}} } \right) \, \\ & \quad + \left( {s_{{{\mathrm{DS}}}} - s_{{{\mathrm{rel}}}} } \right) \\ & = c^{{2}} \cdot s_{{{\mathrm{E}},{\mathrm{DS}}}} \cdot \left[ {1 - \left( {{{s_{{{\mathrm{E}},{\mathrm{rel}}}} } \mathord{\left/ {\vphantom {{s_{{{\mathrm{E}},{\mathrm{rel}}}} } {s_{{{\mathrm{E}},{\mathrm{DS}}}} }}} \right. \kern-0pt} {s_{{{\mathrm{E}},{\mathrm{DS}}}} }}} \right)} \right] \, + s_{{{\mathrm{DS}}}} \cdot \\ & \quad \left[ {1 - \left( {{{s_{{{\mathrm{rel}}}} } \mathord{\left/ {\vphantom {{s_{{{\mathrm{rel}}}} } {s_{{{\mathrm{DS}}}} }}} \right. \kern-0pt} {s_{{{\mathrm{DS}}}} }}} \right)} \right] < < \, 0. \\ \end{aligned} $$

The *s*_neg_ (SI units [J/kg−K]) of plant as DS existing as matter is defined based on specific entropy (entropy density per unit mass of matter). Therefore, the change in entropy density of radiation energy exchanged (entropy per unit energy), is multiplied by, *c*^2^. In the right-hand side of Eq. (4.2):

(i) *s*_neg,rad_ is negentropy build-up of the plant as DS, by the generation of radiation entropy due to release of *Ė*_em_Tleaf_ at *s*_E,rel_.
(ii) *s*_neg,mat_ is negentropy build-up of the plant as DS, by matter exchanged.
(iii) Ratios of entropy densities, (*s*_E,rel_/*s*_E,DS_) and (*s*_rel_/*s*_DS_), correlate with negentropy build-up of DS, for given *s*_E,DS_ and *s*_DS_.

Plants can build their *s*_neg_ also by processing radiation (in addition to matter); where, *s*_neg,rad_ >> *s*_neg,mat_; hence, this study focuses exclusively on *s*_neg,rad_. Equation (4.2) is written for radiation energy exchanged as [using approximation given by Eq. (4.1)],

4.2.1 $$ s_{{{\mathrm{neg}},{\mathrm{rad}}}} = c^{{2}} \cdot s_{{{\mathrm{E}},{\mathrm{DS}}}} \cdot [{1} - \left( {s_{{{\mathrm{E}},{\mathrm{rel}}}} /s_{{{\mathrm{E}},{\mathrm{DS}}}} } \right)] \approx c^{{2}} \cdot s_{{{\mathrm{E}},{\mathrm{DS}}}} \cdot ({1} -\Pi _{{\mathrm{E}}} ,{\mathrm{leaf}}) \approx - c^{{\mathbf{2}}} \cdot (s_{{{\mathbf{E}},{\mathbf{rel}}}} - s_{{{\mathbf{E}},{\mathbf{in}}}} ) \, < < \, {\mathbf{0}}. $$

Change in entropy density of radiation from low *s*_E,in_ to high *s*_E,rel_ by the leaf is amplified by a huge factor (*c*^2^), while determining the negentropy build-up of the plant. Therefore, even a small increase in *s*_E,rel_ and a small reduction in *s*_E,in_ can significantly increase the negentropy build-up of the plant. It is difficult to artificially change the entropy density of radiation (also at laboratory-scale), as is done by a plant-leaf. From Eq. (4.2.1), higher negentropy build-up of plant is by increasing the processing level of absorbed PAR, i.e. by increasing Π_E,leaf_. Possible role of transpiration cooling of plant-leaf in the entropy production in surroundings [32] is not considered in this study. Using Eq. (3.2.1), the magnitude of negentropy build-up of plant is given as,

4.2.2 $$ \left( {|s_{{{\mathrm{neg}},{\mathrm{rad}}}} |/c^{{2}} } \right) = \frac{{\Delta \dot{S}_{{{\mathrm{rad}},{\mathrm{gen}}}} }}{{\dot{E}_{{{\mathrm{em}}\_{\mathrm{Tleaf}}}} }} + s_{{{\mathrm{E}},{\mathrm{in}}}} \cdot \frac{{{\upeta }_{{{\mathrm{ph}},{\mathrm{PAR}}}} }}{{1 - {\upeta }_{{{\mathrm{ph}},{\mathrm{PAR}}}} }}. $$

Part of this negentropy build-up can be made available as the source of free energy of the plant, as per the interpretation of Schroedinger [28]. This part can be stored as plant-growth and then made available as free-energy to the plant (e.g. to do work against Earth’s gravity), which is 2nd term in Eq. (4.2.2). Remaining part of $\left| {s_{{{\mathrm{neg}},{\mathrm{rad}}}} } \right|$ is dissipated in the surroundings [1st term in Eq. (4.2.2)], which is the excess payment of negentropy debt for existence of plant as self-organized DS. Thus, Eq. (4.2.2) gives negentropy build-up of the plant as sum of:

(i) Net radiation entropy generated by the plant-leaf (per unit radiation energy released) in its surroundings, which is the mandatory compliance of 2nd Law.
(ii) Possible plant-growth through the photochemical reaction given by Eq. (3.1.1), by integrating low entropy density PAR in the bonds of C_6_H_12_O_6_.

**Fig. 3:**
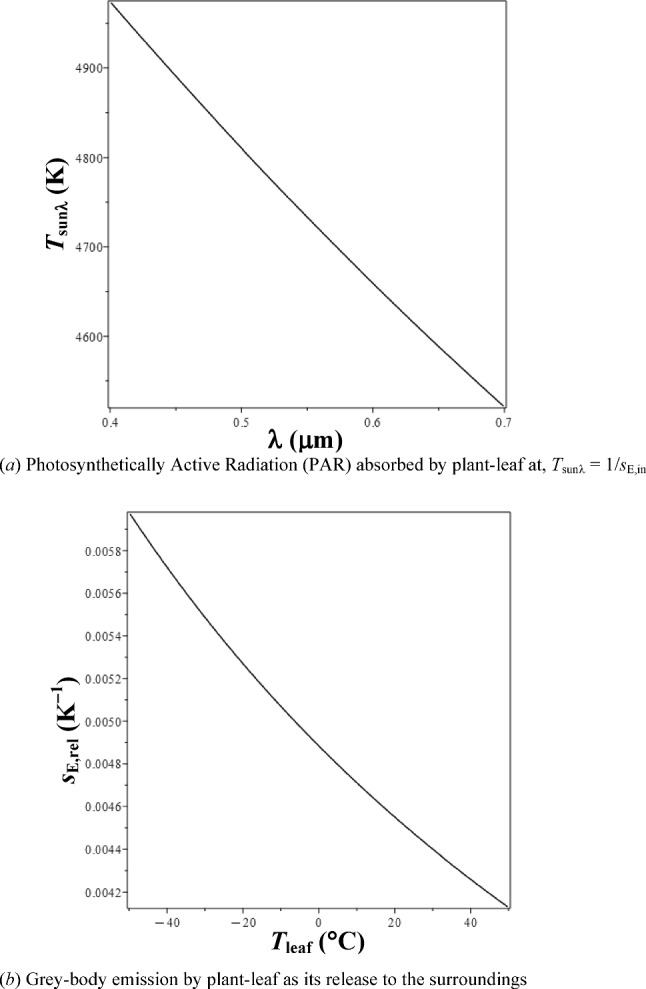
Variation of entropy density of radiation, *s*_E_. **a** Photosynthetically Active Radiation (PAR) absorbed by plant-leaf at,$T_{{{{\mathrm{sun}}}}{\lambda}} = { 1}/s_{{{\mathrm{E}},{\mathrm{in}}}}$. **b** Grey-body emission by plant-leaf as its release to the surroundings

In Eq. (4.2.1), *s*_E,in_ of the absorbed PAR is given as [33], *s*_E,in_ = $\frac{{S_{{\upnu }} }}{{E_{{\upnu }} }}$ = $\frac{k}{h \cdot \nu } \cdot \left[ {\left( {1 + \frac{1}{{{\upxi }_{{{\mathrm{sun}}}} }}} \right) \cdot {\mathrm{ln}}\left( {1 + {\upxi }_{{{\mathrm{sun}}}} } \right) - {\mathrm{ln}}\left( {{\upxi }_{{{\mathrm{sun}}}} } \right)} \right]$; where, $\xi_{{{\mathrm{sun}}}} = \left[ {{\mathrm{exp}}\left( {\frac{h \cdot \nu }{{k \cdot T_{{{\mathrm{sun}}}} }}} \right) - 1} \right]^{ - 1}$. If Λ_sun_(*T*_sun_) = [*h*⋅*c*/(*k*⋅*T*_sun_)] ≈ 2.492746 µm (dimensional constant, *h*: Planck’s const., *k*: Boltzmann’s const.),

4.3 $$  \begin{aligned}   s_{{{\mathrm{E,in}}}} \left( \lambda  \right){\text{ }} =  & \frac{{\left( {\lambda /\Lambda _{{{\mathrm{sun}}}} } \right)}}{{T_{{{\mathrm{sun}}}} }} \cdot \left\{ {\exp \left( {\frac{{\Lambda _{{{\mathrm{sun}}}} }}{\lambda }} \right) \cdot \ln \left[ {\frac{1}{{1 - \exp \left( { - ~\frac{{\Lambda _{{{\mathrm{sun}}}} }}{\lambda }} \right)}}} \right]} \right. \\     & \left. { - \ln \left[ {\frac{1}{{\exp \left( {\frac{{\Lambda _{{{\mathrm{sun}}}} }}{\lambda }} \right) - 1}}} \right]} \right\}; \\  \end{aligned}  $$

where, (Λ_sun_/λ) > 1, in PAR. Spectral temperature of Sun {*T*_sunλ_in [K], Fig. 3a} is the reciprocal of its spectral entropy-density,

4.3.1 $$T_{{{sun{\lambda}}}} ({\lambda }) \, = \, [{1}/s_{{{\mathrm{E}},{\mathrm{in}}}} ({\lambda })]. $$

It represents the Sun’s temperature at a particular λ, for given surface temperature, *T*_sun_ (≈ 5772 K). As shown in Fig. 3a, *T*_sunλ_ decreases with increasing λ (almost linearly) from 4974.5 to 4520.7 K (Δ*T*_sunλ_ ~ 9.123%), in PAR. In Eq. (4.2.1), *s*_E,rel_ of the released spectrum [distributed as black-body radiation at *T*_leaf_ (in [K])] is,

4.4 $$ s_{{{\mathrm{E}},{\mathrm{rel}}}} \left( {T_{{{\mathrm{leaf}}}} } \right) = \frac{4}{3}\frac{{\left( {1 - {\upeta }_{{{\mathrm{ph}},{\mathrm{PAR}}}} } \right) \cdot n \cdot h \cdot \nu }}{{T_{{{\mathrm{leaf}}}} }} \cdot \frac{1}{{\left( {1 - {\upeta }_{{{\mathrm{ph}},{\mathrm{PAR}}}} } \right) \cdot n \cdot h \cdot \nu }} = \frac{4}{{3T_{{{\mathrm{leaf}}}} }} $$

Thus, ratio of the entropic potential of grey-body radiation (emitted at *T*_leaf_) by its energy is inversely proportional to *T*_leaf_ (also inferred from [34]). Figure 3b gives *s*_E,rel_(*T*_leaf_) [>> *s*_E,in_(λ), ref. Eq. (4.1)], which shows that *s*_E,rel_ decreases monotonically from 5.98 × 10^−3^ to 4.128 × 10^−3^ K^−1^ (− $\Delta s_{{{\mathrm{E}},{\mathrm{rel}}}}$ ~ 30.97%), with increasing *T*_leaf_ [= *f*(*T*_a_)] in the range, − 45 to + 55 °C. Therefore, change in* T*_a_ has a greater role in the plant’s $\Pi _{{{\mathrm{E}},{\mathrm{leaf}}}}$, ${\upeta }_{{{\mathrm{ph}},{\mathrm{PAR}}\_{\mathrm{max}}}}$, and $|s_{{{\mathrm{neg}},{\mathrm{rad}}}} |$, relative to λ of sunlight processed in PAR.

**Fig. 4:**
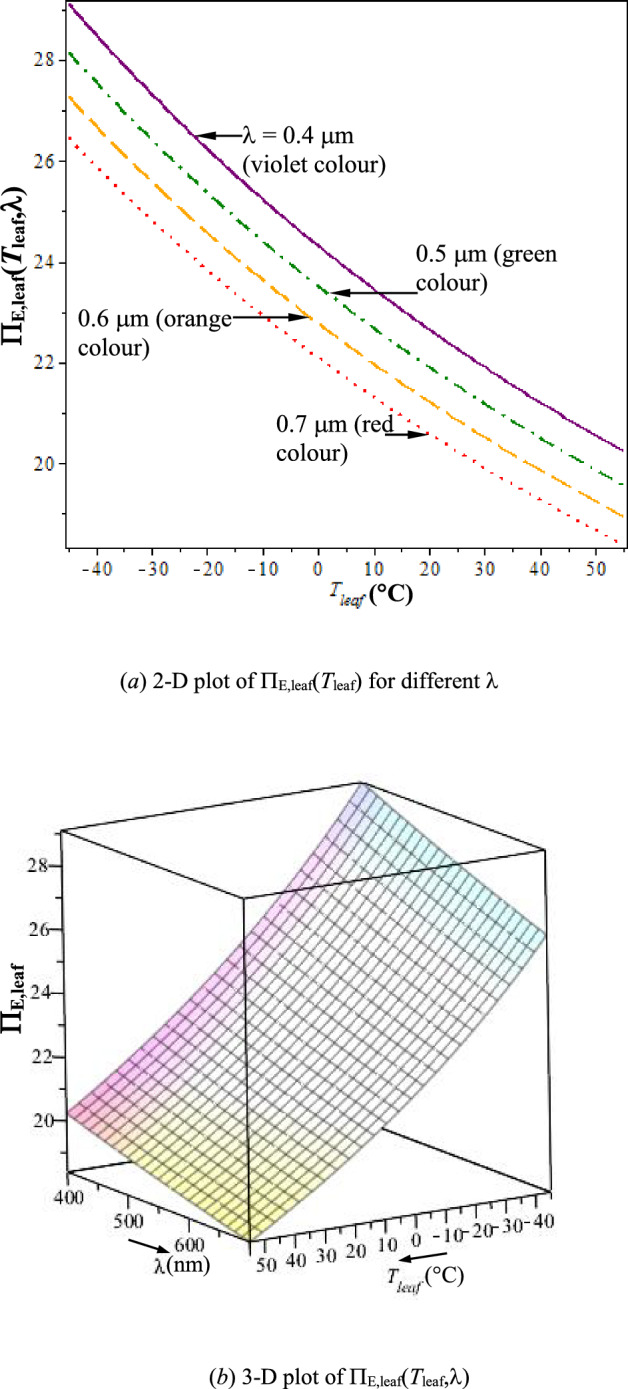
Plant-leaf’s processing level of PAR, $\Pi _{{{\mathrm{E}},{\mathrm{leaf}}}} = f(T_{{{\mathrm{leaf}}}} ,{{\lambda}})$. **a** 2-D plot of $\Pi _{{{\mathrm{E}},{\mathrm{leaf}}}} \left( {T_{{{\mathrm{leaf}}}} } \right)$ for different λ. **b** 3-D plot of $\Pi _{{{\mathrm{E}},{\mathrm{leaf}}}} \left( {T_{{{\mathrm{leaf}}}} ,{{\lambda}}} \right)$

From Eqs. (4.3.1) and (4.4), the ratio,

4.4.1 $$ \begin{aligned}   \Pi _{{{\mathrm{E}},{\mathrm{leaf}}}} \left( {T_{{{\mathrm{leaf}}}} ,{{\lambda}}} \right) =  & \left[ {s_{{{\mathrm{E}},{\mathrm{rel}}}} \left( {T_{{{\mathrm{leaf}}}} } \right)/s_{{{\mathrm{E}},{\mathrm{in}}}} ({{\lambda}})} \right] \\     =  & \left( {4/3} \right)\left( {T_{{{{\mathrm{sun}}}}\lambda} /T_{{{\mathrm{leaf}}}} } \right); \\  \end{aligned}  $$

is in Figs. 4a, b. It shows that, *s*_E,rel_ >> *s*_E,in_ (Π_E,leaf_ ~ 20), which leads to the negentropy build-up of the plant as self-organizing DS [ref. Eq. (4.2.1)]. From the inequality in Eq. (3.2.1), Π_E,leaf_ (>> 1) gives enough margin for the sustenance and negentropy build-up of plant, with additional possibility of growth (limited by η_ph,PAR_max_). From Fig. 4, Π_E,leaf_ is seen to be a stronger function of *T*_leaf_ than λ in PAR and from Eq. (4.4.1), Π_E,leaf_ ∝ (1/*T*_leaf_) for given λ. Thus,

(i) ***Higher processing level of the plant-leaf is of blue colour, followed by green and then red colour***. Balegh & Biddulph [35] showed that green light once absorbed drives photosynthesis with higher ${\upeta }_{{{\mathrm{ph}}}}$ than red-light. This reported observation agrees with the thermodynamic basis of increasing Π_E,leaf_ with decreasing λ, shown in Fig. 4a, i.e. green-light has higher Π_E,leaf_ than red (& lower than blue-light). Measured absorptance spectra showed that ordinary green leaves of land plants absorb a substantial fraction of green light [36].
(ii) ***Higher processing level of the plant-leaf is at lower T***_**leaf**_. Lower *T*_leaf_ (at lower ambient temperature, *T*_a_, or by transpiration cooling of plant-leaf for given *T*_a_) facilitates the processing of PAR. That the spectral quantum yield increases with decreasing *T*_leaf_ is also reported by McCree [6], based on experimental studies on oat leaf.

**Fig. 5:**
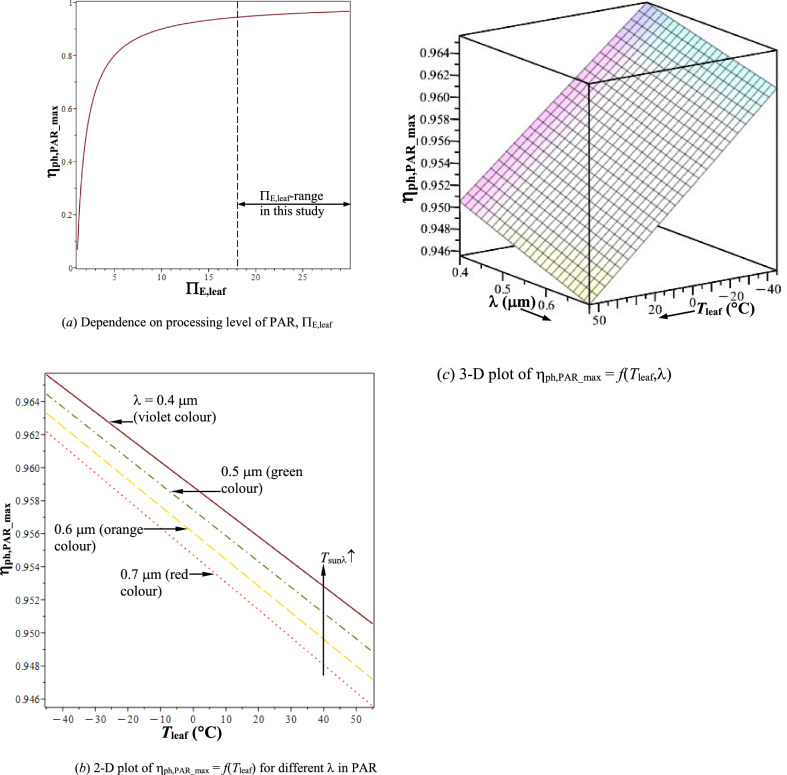
Maximum photosynthetic efficiency, ${\upeta }_{{{\mathrm{ph}},{\mathrm{PAR}}\_{\mathrm{max}}}}$. **a** Dependence on processing level of PAR, $\Pi _{{{\mathrm{E}},{\mathrm{leaf}}}}$. **b** 2-D plot of ${\upeta }_{{{\mathrm{ph}},{\mathrm{PAR}}\_{\mathrm{max}}}} = f\left( {T_{{{\mathrm{leaf}}}} } \right)$ for different λ in PAR. **c** 3-D plot of ${\upeta }_{{{\mathrm{ph}},{\mathrm{PAR}}\_{\mathrm{max}}}} = f\left( {T_{{{\mathrm{leaf}}}} ,{{\lambda}}} \right)$

Figure 5a shows that ${\upeta }_{{{\mathrm{ph}},{\mathrm{PAR}}\_{\mathrm{max}}}} = f(\Pi _{{{\mathrm{E}},{\mathrm{leaf}}}} )$ [Eq. (3.1.3)], increases monotonically from 0 to 0.967 with Π_E,leaf_ increasing from 1 to 30. It also shows the range of Π_E,leaf_ in this study from 18.39 to 29.12, which corresponds to η_ph,PAR_max_ varying from, 0.945584—0.965625 (ref. Table 2). Qualitative trends of ${\upeta }_{{{\mathrm{ph}},{\mathrm{PAR}}\_{\mathrm{max}}}} = f(T_{{{\mathrm{leaf}}}} ,{{\lambda}})$ in Figs. 5b,c are similar to those of Π_E,leaf_(*T*_leaf_,λ) in Figs. 4a,b. From Eqs. (3.1.3), (4.3.1), and (4.4), linear relation between η_ph,PAR_max_ and *T*_leaf_ is obtained as;

4.5 $$ {\upeta} _{{{\mathrm{ph,PAR\_max}}}}  = 1 - \left( {{3 \mathord{\left/ {\vphantom {3 4}} \right. \kern-\nulldelimiterspace} 4}} \right) \cdot \left( {{{T_{{\mathrm{leaf}}} } \mathord{\left/ {\vphantom {{T_{\mathrm{leaf}}} {T_{\mathrm{sunl}} }}} \right. \kern-\nulldelimiterspace} {T_{{\mathrm{sun}}\lambda} }}} \right).  $$

**Table 2:** Highest and lowest thermodynamic performance of plant-leaf in PAR in feasible *T*_leaf_-range

Performance parameter (Φ)	$\Pi _{{{\mathrm{E}},{\mathrm{leaf}}}}$	${{\left| {s_{{{\mathrm{neg}},{\mathrm{rad}}}} } \right|} \mathord{\left/ {\vphantom {{\left| {s_{{{\mathrm{neg}},{\mathrm{rad}}}} } \right|} {c^{{2}} }}} \right. \kern-0pt} {c^{{2}} }}$	${\upeta }_{{{\mathrm{ph}},{\mathrm{PAR}}\_{\mathrm{max}}}}$
*T*_leaf_ = + 55 °C, λ = 0.7 μm; *Lowest Performance*:	18.39	3.844 × 10^−3^ K^−1^	0.945584
*T*_leaf_ = − 45 °C, λ = 0.4 μm; *Highest Performance*:	29.12	5.647 × 10^−3^ K^−1^	0.965625
[(Φ_max_ − Φ_min_)/Φ_min_] × 100%	58.35	46.90	2.119

With increasing *T*_leaf_ [for given *T*_sunλ_(λ)], η_ph,PAR_max_ decreases linearly; and for increasing λ in PAR from 0.4 to 0.7 μm, η_ph,PAR_max_ decreases [for given *T*_leaf_, since, *T*_sunλ_(λ) decreases]. Therefore, upper limit to plant-growth is raised at lower *T*_leaf_ and by the leaf’s ability to process lower λ in PAR. Rate of sugar production correlates with increase in plant-growth [limited by η_ph,PAR_max_ in Eq. (4.5)], which is higher at low *T*_leaf_ [24]. Results in Figs. 4, 5 are qualitatively (in trend) analogous to the Carnot cycle model [37]. Maximum thermodynamic cycle efficiency is that of a reversible Carnot engine, which does not generate net entropy in the surroundings. Efficiency of ideal Carnot cycle operating between heat source at absolute temperature, *T*_source_, and heat sink at absolute temperature, *T*_sink_, is given as;

4.5.1 $$ {\upeta }_{{{\mathrm{cycle}}\_{\mathrm{max}}}} = \, {1} - \left( {T_{{{\mathrm{sink}}}} /T_{{{\mathrm{source}}}} } \right). $$

Comparing Eqs. (4.5) and (4.5.1): (i) *T*_sunλ_ is analogous to *T*_source_; (ii) *T*_leaf_ is analogous to *T*_sink_.

From Eqs. (4.2.1), (4.3.1), and (4.4),

4.6 $$\begin{aligned} &s_{{{\mathrm{neg}},{\mathrm{rad}}}} = - c^{{2}} \times \left( {\frac{4}{{3T_{{{\mathrm{leaf}}}} }} - \frac{1}{{T_{{{\mathrm{sun}}\lambda}} }}}\right); {\text{ i}}.{\mathrm{e}}. \\&\left( {{{\left| {s_{{{\mathrm{neg}},{\mathrm{rad}}}} } \right|}/{c^{{2}} }}} \right)= \left( {\frac{4}{{3T_{{{\mathrm{leaf}}}} }} -\frac{1}{{T_{{{\mathrm{sun}}\lambda}} }}} \right). \end{aligned}$$

**Fig. 6:**
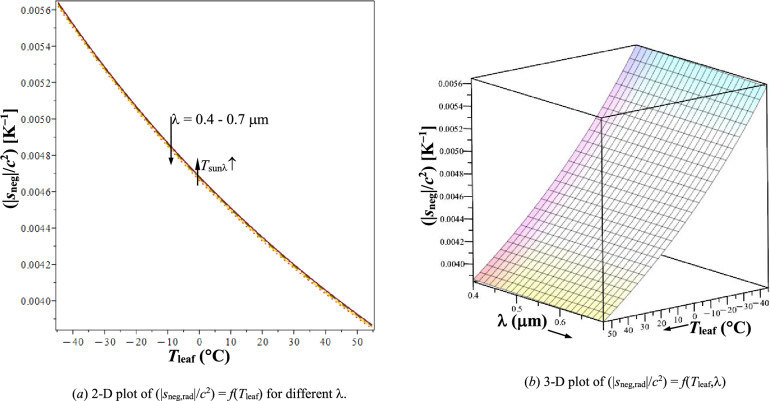
Variation of $\left( {\left| {s_{{{\mathrm{neg}},{\mathrm{rad}}}} } \right|/c^{{2}} } \right)$ with *T*_leaf_ and λ in PAR. **a** 2-D plot of $\left( {\left| {s_{{{\mathrm{neg}},{\mathrm{rad}}}} } \right|/c^{{2}} } \right) = f\left( {T_{{{\mathrm{leaf}}}} } \right)$ for different λ. **b** 3-D plot of $\left( {\left| {s_{{{\mathrm{neg}},{\mathrm{rad}}}} } \right|/c^{{2}} } \right) \, = f\left( {T_{{{\mathrm{leaf}}}} ,{{\lambda}}} \right)$

Negentropy build-up of the plant as dissipative structure (DS) is determined by the difference between Sun’s spectral temperature (*T*_sunλ_) and its leaf’s temperature, *T*_leaf_. Figure 6 give the variation of ($\left| {s_{{{\mathrm{neg}},{\mathrm{rad}}}} } \right|$/*c*^2^) with *T*_leaf_ and λ in PAR as per Eq. (4.6), which shows its increase with decreasing *T*_leaf_ and weak dependence on λ (though $\left| {s_{{{\mathrm{neg}},{\mathrm{rad}}}} } \right|$ is somewhat higher at lower λ). From Eqs. (4.5) and (4.6), relation between maximum plant-growth and negentropy build-up is obtained as,

4.6.1 $$ {\upeta }_{{{\mathrm{ph}},{\mathrm{PAR}}\_{\mathrm{max}}}} = \frac{{T_{{{\mathrm{sun}}\lambda}} }}{{T_{{{\mathrm{sun}}\lambda}} + \left( {\frac{{\left| {s_{{{\mathrm{neg}},{\mathrm{rad}}}} } \right|}}{{c^{2} }}} \right)^{ - 1} }} $$

Since *T*_sunλ_ is fixed, increase in ($\left| {s_{{{\mathrm{neg}},{\mathrm{rad}}}} } \right|$/*c*^2^) results in increase in η_ph,PAR_max_, both are higher at lower *T*_leaf_. For the range, *T*_leaf_ =  − 45 to 55 °C and λ = 0.4–0.7 μm, highest and lowest performance parameters are in Table 2, which are the diagonally opposite corners in Figs. 4b, 5c, and 6b. Increase from lowest to highest is the most for Π_E,leaf_ (processing level, 58.35%); followed by $\left| {s_{{{\mathrm{neg}},{\mathrm{rad}}}} } \right|$ (negentropy build-up, 46.9%); and then η_ph,PAR_max_ (maximum growth potential, 2.119%). These quantitative estimates highlight the dominant purpose of processing of PAR by leaf for radiation entropy generation in the surroundings for mandatory 2nd Law compliance, rather than for possible plant-growth.

## Conclusions

(i) Plant functions as a self-organized dissipative structure (DS), because its leaf generates net radiation entropy in the surroundings for existence of plant with possible growth. Radiation entropy production in the surroundings by plant-leaf is requirement for mandatory 2nd Law compliance, for the photochemical reaction to take place leading to the formation of glucose.
(ii) Radiation entropy production is a simple task for any green plant but is not yet realised artificially (even at laboratory-scale), due to the difficulty in achieving the high processing level of PAR. Entropy density (entropy-energy ratio) of Photosynthetically Active Radiation (PAR) that is processed and then emitted by the plant-leaf (*s*_E,rel_) exceeds that absorbed (*s*_E,in_), by a factor, Π_E,leaf_ = (*s*_E,rel_/*s*_E,in_) ~ 20.
(iii) The Π_E,leaf_ >> 1, is an entropic measure of processing level of PAR by the plant-leaf. It determines the margin for plant-growth [Eq. (3.2.2)] based on photosynthetic efficiency in PAR, ${\upeta }_{{{\mathrm{ph}},{\mathrm{PAR}}}}$ (> 0, for plant-growth). The Π_E,leaf_ is a stronger function of the leaf’s temperature (higher at lower *T*_leaf_) relative to the wavelength within PAR processed (higher at lower λ).
(iv) Dimensionless net radiation entropy generated by the plant-leaf in its surroundings, Π_Srad,gen_, is introduced; where, Π_Srad,gen_ > 0, must be satisfied for mandatory 2nd Law compliance. The Π_Srad,gen_ is the net radiation entropy generated in surroundings per unit radiation entropy absorbed for plant-growth. For, Π_Srad,gen_ = 0, plant-growth is maximum; and for, $\Pi_{\mathrm{Srad,gen}} \to \infty$, there is no plant-growth.
(v) Plants can build their negentropy (*s*_neg_ < 0) also by processing radiation (in addition to matter), which has a much higher contribution to their self-organization with growth potential. In the negentropy build-up of plant by radiation entropy generation (*s*_neg,rad_ < 0), changes in *s*_E,rel_ and *s*_E,in_ are multiplied by a huge factor, *c*^2^; where, *c* is the speed of light. Hence, even a small rise in *s*_E,rel_ and a small drop in *s*_E,in_ can significantly increase the magnitude of the negentropy build-up of plant by radiation entropy generation, $\left| {s_{{{\mathrm{neg}},{\mathrm{rad}}}} } \right|$; which increases with decreasing *T*_leaf_ and is higher at lower wavelength (in PAR) processed.
(vi) Part of $\left| {s_{{{\mathrm{neg}},{\mathrm{rad}}}} } \right|$ can be the source of free energy of plant; since, it can be stored as plant-growth. Remaining part of $\left| {s_{{{\mathrm{neg}},{\mathrm{rad}}}} } \right|$ is dissipated in the surroundings, which is the excess payment of negentropy debt as part of mandatory 2nd Law compliance for the existence of plant as self-organized DS.
(vii) The η_ph,PAR_max_ is obtained [Eq. (4.5)] in terms of *T*_leaf_ and the spectral temperature of the Sun, $T_{{{\mathrm{sun}}\lambda}} [ = \, ({1}/s_{{{\mathrm{E}},{\mathrm{in}}}} )]$. Expression for ${\upeta }_{{{\mathrm{ph}},{\mathrm{PAR}}\_{\mathrm{max}}}}$ correlates with that for the maximum thermodynamic efficiency of reversible Carnot engine, ${\upeta }_{{{\mathrm{cycle}}\_{\mathrm{max}}}}$. For both, ${\upeta }_{{{\mathrm{ph}},{\mathrm{PAR}}\_{\mathrm{max}}}}$ and ${\upeta }_{{{\mathrm{cycle}}\_{\mathrm{max}}}}$, no net entropy is generated in the surroundings.

## Data Availability

This manuscript has no associated data. [Authors’ comment: This is a theoretical study, and no experimental data has been listed.]
